# Antibiotic resistance patterns of *Staphylococcus aureus*, *Escherichia coli*, *Salmonella*, *Shigella* and *Vibrio* isolated from chicken, pork, buffalo and goat meat in eastern Nepal

**DOI:** 10.1186/s13104-019-4798-7

**Published:** 2019-11-21

**Authors:** Kamana Bantawa, Shiv Nandan Sah, Dhiren Subba Limbu, Prince Subba, Arjun Ghimire

**Affiliations:** Department of Microbiology, Central Campus of Technology, Tribhuvan University, Dharan, Nepal

**Keywords:** *S. aureus*, *E. coli*, *Salmonella*, *Shigella*, *Vibrio*, Antibiotic resistance

## Abstract

**Objective:**

Food-borne pathogens are a major cause of illnesses, death and expenses. Their occurrence in meat and other food is considered a global health problem. The burden of food-borne disease is increasing due to antimicrobial resistance which represents a greater risk of treatment failure. However, very little is known about the antibiotic resistance profile of food-borne pathogens in Nepal. This study was conducted to examine the antibiotic resistance profile of common food-borne bacterial pathogens isolated from raw meat sold in Nepal. A total of 83 meat samples were collected from the market and analyzed.

**Results:**

The prevalence of *Staphylococcus aureus*, *Escherichia coli*, *Salmonella*, *Shigella,* and *Vibrio* were 68%, 53%, 35%, 6%, and 6% respectively. The resistance of *Salmonella* was most frequently observed to amoxicillin (100%), tetracycline (24%), chloramphenicol (11%), and nalidixic acid (11%). *S. aureus* was resistant to amoxicillin (100%) followed by tetracycline (63%), nalidixic acid (17%), and cefotaxime (13%) respectively. *Vibrio* isolates resisted amoxicillin (100%), tetracycline (40%) and chloramphenicol (20%). *Shigella* expressed the highest resistance to amoxicillin (100%), followed by chloramphenicol (80%), tetracycline (60%) and nalidixic acid (20%). *E. coli* exhibited the highest resistance to amoxicillin (100%), followed by tetracycline (93%), nalidixic acid (25%) and cefotaxime (19%).

## Introduction

Food-borne pathogens have been the paramount cause of illness and death in the world [1]. As they affect the health and economy, the awareness on food-borne pathogens is increasing [2, 3]. Poultry and other meats also occupy one of the most important reservoirs for pathogenic bacteria [4]. Normally, the meat of healthy animals contains very few or nil microorganisms but contamination arise from slaughtering, transportation and processing [4, 5]. The most important food-borne bacteria transmitted through meat include *Salmonella*, *Shigella*, *Staphylococcus aureus*, *Escherichia coli*, *Campylobacter jejuni*, *Listeria monocytogenes*, *Clostridium perfringens*, *Yersinia enterocolitica* and *Aeromonas hydrophila* [5]. These bacteria usually cause self-limiting gastroenteritis however, invasive diseases and various complexities also may occur. *E. coli* can cause bloody diarrhoea and hemolytic uremic syndrome, *Salmonella* can cause systemic salmonellosis, *S. aureus* is responsible for causing food poisoning, *Shigella* can cause dysentery and *Vibrio* can cause cholera if undercooked meat is consumed [6–8].

With the emergence of bacteria, the consumption of antibiotics is increased by approximately 40% in a decade but their resistance has been becoming a global threat [9]. Apart from clinical settings, large amounts of antibiotics are used in agriculture, food industry, animal husbandry and aquaculture [10]. The use of broad-spectrum antibiotics creates a selective pressure on the bacterial flora, thus increasing the emergence of new antibiotic-resistant bacteria [11, 12]. Similarly, due to incomplete metabolism, unused antibiotics spread to the environment eliciting bacterial adaptation response to develop antibiotic-resistant genes [13, 14]. Antibiotic resistance in bacterial pathogens is higher in poultry, pig and other meat animals [15]. As a result, antibiotic-resistant strains from the gut may contaminate meat during slaughtering and resistant bacteria can infect consumers via meat [16–18]. The transfer of resistant bacteria from meat to humans has been reported in various countries [19–21]. In the transfer of antibiotic-resistant genes from food micro flora to pathogenic bacteria, meat can play an important role [22, 23]. In Nepal, consumption of meat has been increasing but Nepalese butchers and consumers are unaware of food safety [24, 25]. On the other hand, the prevalence of antibiotic-resistant bacteria is increasing due to the haphazard use of antibiotics in human therapy, animal farming and other prophylactic usages [26]. It is difficult to treat the infections caused by multi-drug resistant bacteria as compared to normal bacteria and such strains are of great concern [26]. However, very few studies have been done in Nepal about food-borne bacteria and their antibiotic resistance profile. So, this study aimed to investigate the antibiotic resistance pattern of bacterial isolates from marketed meat in Dharan, eastern Nepal.

## Main text

### Methods

#### Study site

**Fig. 1:**
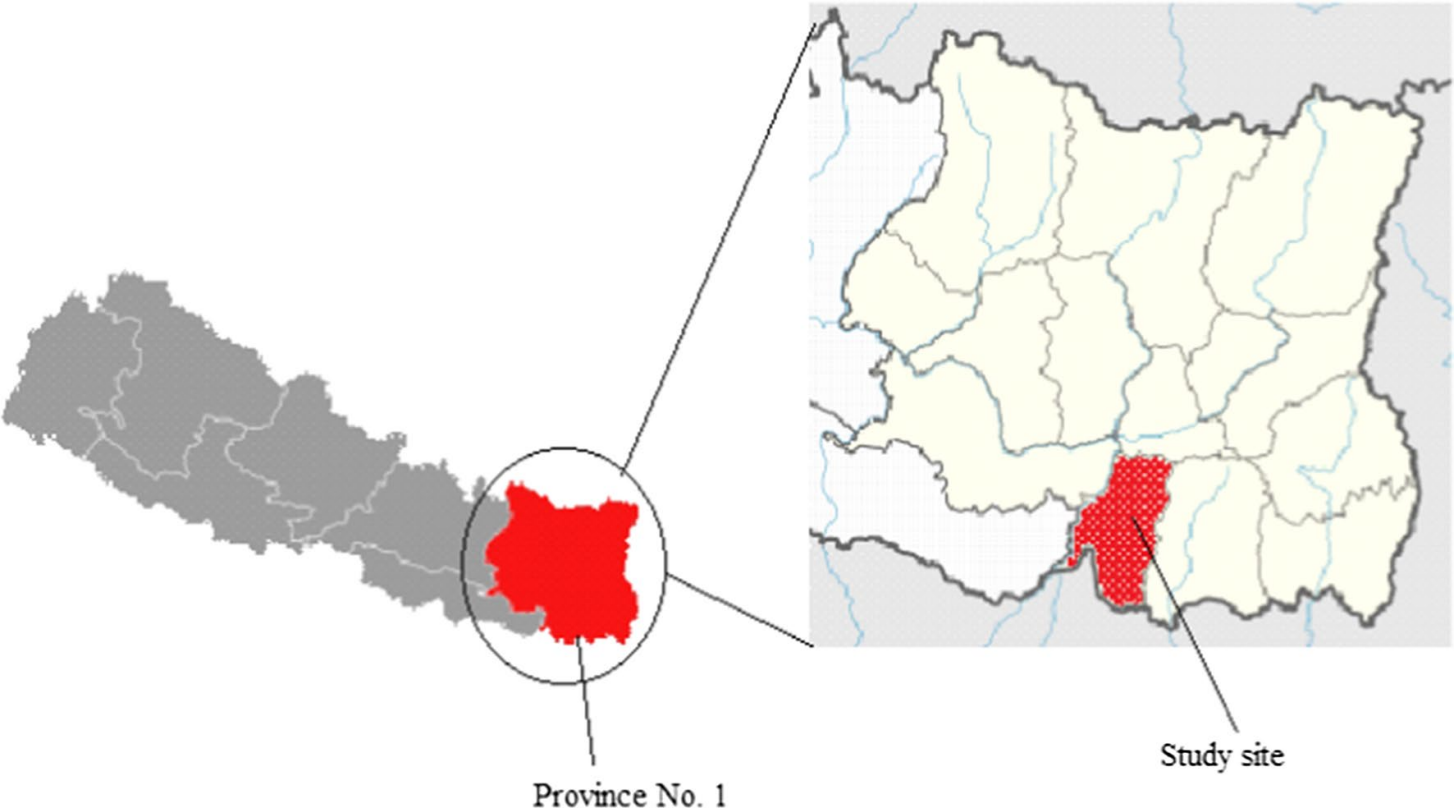
Area of study

The study site is a town of Province number 1 of Nepal (Fig. 1) locating between hills and Terai region at the altitude of 349 m.

#### Analytical methods

A total of 83 (33 chicken, 27 pork, 13 buffalo and 10 goat) meat samples were collected in a UV sterilized zipped plastic bag and transported to the laboratory in an icebox. The samples were collected 6–9 a.m. and processed within 2 h otherwise preserved at 4 °C. To prevent cross-contamination during sampling, sterile gloves and forceps were used. The meat shops are randomly scattered in different locations of Dharan and only one type of meat was collected in a single day.

Isolation of *E*. *coli*, *Salmonella*, *Shigella*, *Vibrio*, and *S. aureus* was done by following U.S. FDA guideline [27] in triplicate. The isolates were identified by cultural characteristics, Gram staining, and biochemical tests as described by Bergey’s Manual of Determinative Bacteriology [28]. All of the culture media, reagents and antibiotics were purchased from Himedia, India. The significant association of dependent variables was determined by Chi-square test at 5% level of significance by using software SPSS version 16.

Antibiotic susceptibility test (AST) of all identified isolates was done on eight antibiotics following Kirby Bauer disc diffusion method. The interpretation of sensitive (S), intermediate sensitive (I) and resistant (R) were done according to the CLSI guidelines [29]. Selection of antibiotics was based on the common antibiotics used in Nepal and those recommended by the World Health Organization (WHO) for routine integrated antimicrobial resistance monitoring [30]. These antibiotics were amoxicillin (Amx 10 µg), azithromycin (Azm 15 µg), amikacin (Ak 30 µg), cefotaxime (Ctx 30 µg), nalidixic acid (NA 30 µg), ciprofloxacin (Cip 30 µg), tetracycline (TE 30 µg) and chloramphenicol (C 30 µg). For quality control, ATCC cultures of *E. coli* 25922, *S. aureus* 25923, *Salmonella* 35664, *Shigella* 23354 and *Vibrio* 39315 were used.

### Results and discussions

**Fig. 2:**
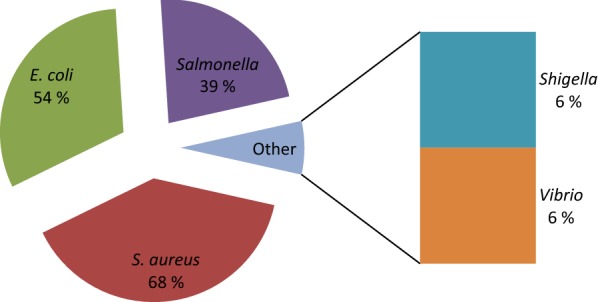
Prevalence of bacterial isolates

**Fig. 3:**
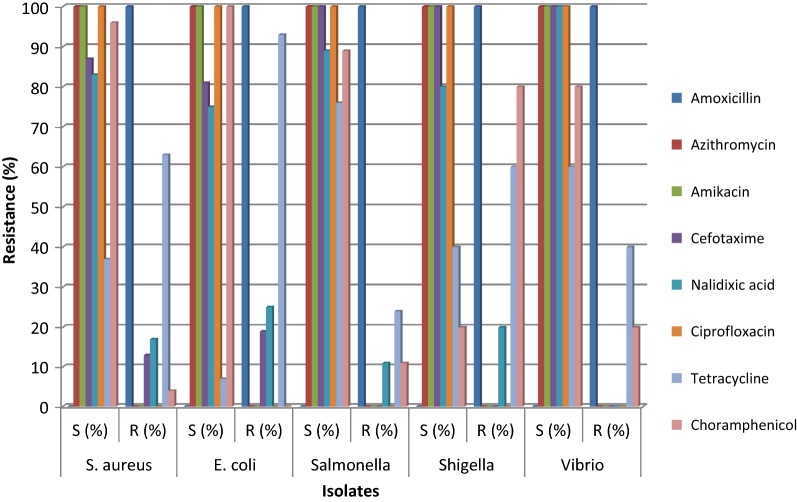
Antibiotic resistance patterns of bacterial isolates

Resistance enables bacteria to escape from being killed by antibiotics and reduces the ability to treat infections [31]. Therefore, antibiotics resistance has been considered one of the greatest threats to medicine [32]. Meat also plays an important role in the transfer of antibiotics resistant genes in term of antibiotic residues. The prevalence of bacterial isolates is shown in Fig. 2 and their antibiotic resistance profiles are tabulated in Fig. 3. The significant association was not seen between the type of meat and presence of *S. aureus*, *Shigella*, *Vibrio* and *E*. *coli* (p > 0.05) but observed between type of meat and *Salmonella* (p < 0.05). It shows that chicken meat may harbour the more *Salmonella* than other type of meat which may come from faecal matters during processing. The prevalence of bacterial isolates was found to be higher than previous studies in Ethiopia, China and Greece [33–35]. The reason behind the higher prevalence rates could be related to the difference in time, place and season of research. Furthermore, higher prevalence rates might be due to unhygienic processing, improper cleaning, deficient handling, and post-processing contamination from the polluted environment.

Although *S. aureus* and its antimicrobial resistance pattern have been extensively studied in livestock and foods in other countries, limited studies have been done in Nepal. Our result reported the higher resistance rates of isolates than in studies of other countries like Ethiopia, Thailand, China, and South Africa [33, 36–38]. The increased resistance of isolates against commonly used antibiotics may be due to the indiscriminate use of common antibiotics. In the case of *E. coli*, neither of them was susceptible to all antibiotics tested. The higher resistance to amoxicillin (100%) tetracycline (93%) and nalidixic acid (25%) was observed. Our result is in agreement with findings of similar studies by in Greece, Canada and Jamaica [35, 39, 40]. But, other studies by Atnafie et al. and Van et al. do not support our findings [41, 42]. The variation on the rate of resistance can be related to the difference in time and place. Another reason for the difference in resistance rates might be a rapid change in antibiotic sensitivity patterns of bacteria within a short period.

In this study, all isolates of *Salmonella* developed resistance against amoxicillin (100%) followed by tetracycline (24%), nalidixic acid (11%) and chloramphenicol (11%), Studies in other countries show highly variable results. For instance, a study by Akbar et al. [36] reported that 73% *Salmonella* isolates were resistant to tetracycline, 18.48% resistant to chloramphenicol, 36% to nalidixic acid and 27% resistant to ciprofloxacin. Similarly, Odoch et al. [43] reported 50% isolates were resistant to ciprofloxacin, 5.1% to tetracycline and 5.1% to chloramphenicol. The reason behind the variation in resistance rates could be related to the difference in antibiotics usage along with place and season of research. Osaili et al. reported all of the *Salmonella* isolates were resistant to one or more antibiotics and the majority of isolates were sensitive to most of the tested antibiotics [44] which complies with our findings. Our findings were similar to the antibiotic resistance pattern of *Salmonella* isolated from human suffering from diarrhoea and other enteric infections [45–47]. The similarity in the resistance pattern could be due to the usage of similar antibiotics in human and veterinary medicine.

Out of the total *Shigella* isolates, 100%, 80%, 60%, and 20% of them were resistant to amoxicillin, chloramphenicol, tetracycline and nalidixic acid respectively. Our results were higher than the similar study by Garedew et al. [48] which reported 46.9% and 9.4% isolates resistant to amoxicillin and tetracycline respectively. The higher resistance rates to different antibiotics in Nepal could be due to the excessive use and irrational prescription of antibiotics. In a study by Debas et al., all of the isolates were found sensitive to ciprofloxacin [49] which is similar to our study. The resistance pattern of *Shigella* isolates was in companion to some previous studies in human samples [45–47]. This resemblance might be due to the common prescription of antibiotics to human and veterinary medicine. Amoxicillin was resisted by all *Vibrio* isolates while a moderate rate of resistance was observed for tetracycline (40%). The resistance profile of *Vibrio* isolates was analogous to the previous study in seafood [50] but greater than the study on retail shrimps in Vietnam which reported only 24.6% resistant to tetracycline [51]. This difference in resistance profile could be correlated to time and place of study as the frequency of resistance varies from time to time and place to place. Our results demand the periodic evaluation of the resistance pattern of pathogens to overcome the growth of antibiotic resistance.

### Conclusions

The marketed raw meat of eastern Nepal was highly contaminated with antibiotic-resistant *S*. *aureus*, *E*. *coli*, *Salmonella*, *Shigella*, and *Vibrio*. All of the bacterial isolates expressed resistance towards amoxicillin. The higher resistance was observed against tetracycline and chloramphenicol. So, azithromycin, ciprofloxacin and amikacin should be preferred for the treatment of food-borne bacteria if they are suspected of animal origin. Prudent use of antibiotics should be followed by veterinarians and human medical practitioners. Contamination of meat with intestinal content during slaughtering and other cross contaminations should be minimized. Periodic evaluation of the resistance pattern of pathogens is essential. Routine monitoring of slaughtering conditions, awareness campaign and good kitchen practice should be promoted. Further characterization of isolated pathogens should be done to determine antimicrobial resistance genes and their transfer.

## Limitations

Confirmation of bacteria by molecular methods was not performed. Species identification of *Salmonella*, *Shigella* and *Vibrio* was not done. Similarly, serotyping of *Salmonella* was not done.

## Data Availability

All the required data and materials are provided in the manuscript.

## Abbreviations

AST: antibiotic susceptibility test
ATCC: American Type Culture Collection
CLSI: Clinical and Laboratory Standard Institute
FDA: Food and Drug Administration
SPSS: Statistical Programme for Social Sciences
WHO: World Health Organization
